# Supplementary material for: Humoral and cellular immune responses to COVID-19 mRNA vaccines in immunosuppressed liver transplant recipients
Source: Commun Med (Lond). 2024 Feb 26;4:30. doi: 10.1038/s43856-024-00448-4 (PMC10897323; doi:10.1038/s43856-024-00448-4)
Supplement: Supplementary file 2 — Supplementary Information [file 43856_2024_448_MOESM2_ESM.docx]

**Humoral and cellular immune responses to COVID-19 mRNA vaccines in immunosuppressed liver transplant recipients**

Takuto Nogimori^1,+^, Yuta Nagatsuka^1,2+^, Shogo Kobayashi^2,^*, Hirotomo Murakami^1,2^, Yuji Masuta^1^, Koichiro Suzuki^3^, Yoshito Tomimaru^2^, Takehiro Noda^2^, Hirofumi Akita^1,4,5^, Shokichi Takahama^1^, Yasuo Yoshioka^3^, Yuichiro Doki^2^, Hidetoshi Eguchi^2^ and Takuya Yamamoto^1,5,6,7,8^*

^1^ Laboratory of Precision Immunology, Center for Intractable Diseases and ImmunoGenomics, National Institutes of Biomedical Innovation, Health and Nutrition, Osaka 567-0085, Japan

^2^ Department of Gastroenterological Surgery, Graduate School of Medicine, Osaka University, Osaka, 565-0871, Japan

^3^ The Research Foundation for Microbial Diseases of Osaka University (BIKEN), Osaka, 565-0871, Japan

^4^ Department of Gastroenterological Surgery, Osaka International Cancer Institute, Osaka, 540-0008, Japan.

^5^ Laboratory of Translational Cancer Immunology and Biology, Next-generation Precision Medicine Research Center, Osaka International Cancer Institute, Osaka, 540-0008, Japan

^6^ Department of Virology and Immunology, Graduate School of Medicine, Osaka University, Osaka, 565-0871, Japan

^7^ Laboratory of Aging and Immune Regulation, Graduate School of Pharmaceutical Sciences, Osaka University, Osaka, 565-0871, Japan

^8^ Lead contact

^+^ These authors contributed equally.

*** Corresponding authors:**

Takuya Yamamoto, Ph.D.

Laboratory of Precision Immunology, Center for Intractable Diseases and ImmunoGenomics, National Institutes of Biomedical Innovation, Health and Nutrition, 7-6-8, Saito-Asagi, Ibaraki City, Osaka 567-0085, Japan

Tel: +81-72-641-9819

Fax: +81-72-641-9812

Email: [yamamotot2@nibiohn.go.jp](mailto:yamamotot2@nibiohn.go.jp)

Shogo Kobayashi, M.D., Ph.D., FACS

Department of Gastroenterological Surgery, Graduate School of Medicine, Osaka University

2-2, Yamadaoka, Suita City, Osaka 565-0871, Japan

Phone: +81-6-6879-3251

Fax: +81-6-6879-3259

E-mail: skobayashi@gesurg.med.osaka-u.ac.jp

**Supplementary Figure 1. Antibody responses over time in healthy donors and LTRs stratified into subgroups.**

(a) Anti-RBD IgG endpoint titers over time in plasma samples from vaccinated healthy donors (black), recipients from living donors (red), and recipients from a cadaver (blue) (Sample size, 44 vs 46 vs 8). (b) Anti-RBD IgG endpoint titers over time in plasma samples from vaccinated healthy donors (black), recipients more than 12 years after transplantation (red), and recipients less than 12 years after transplantation (blue) (Sample size, 44 vs 27 vs 27). (c) Anti-RBD IgG endpoint titers over time in plasma samples from vaccinated healthy donors (black), recipients without rejection after transplantation (red), and recipients with rejection after transplantation (blue) (Sample size, 44 vs 38 vs 16). (d) Anti-RBD IgG endpoint titers over time in plasma samples from vaccinated healthy donors (black), recipients without MMF (red), and recipients with MMF (blue) (Sample size, 44 vs 37 vs 17). *P*-values (two-sided) were calculated using the nonparametric Mann-Whitney *U*-test. All experiments were performed once.

**Supplementary Figure 2. Gating strategy for antigen-specific CD4^+^ and CD8^+^ T-cells.**

(a) After gating live single T-cells, based on forward scatter area and height (FSC-A and -H), side scatter area (SSC-A), live/dead cell exclusion, and CD3 staining, we separated the peripheral blood mononuclear cells (PBMCs) into CD4^+^ and CD8^+^ T-cells. Subsequently, CD4^+^ and CD8^+^ T-cells were further divided into memory phenotypes based on the expression of CD27 and CD45RO. For spike-specific CD4^+^ T-cells, memory cells were gated based on the expression of CD154. We defined CD154^+^CD4^+^ T-cells expressing IFN-γ, TNF, or IL-2 as Th1 cells and expressing IL4 or IL-13 as Th2 cells. (b) The frequency of spike-specific CD154^+^CD4^+^ T-cells in CD4^+^ total memory T-cells over time from vaccinated healthy donors (black), CNI group (red), and CNI+other drug(s) group (blue). *P*-values (two-sided) were calculated using the Wilcoxon matched-pairs signed rank test. Sample size, 1m after 2nd: 23 vs 17 vs 26, 3m after 2nd: 22 vs 16 vs 22, 6m after 2nd: 43 vs 21 vs 29, 1m after 3rd: 43 vs 20 vs 29. All experiments were performed once.

**Supplementary Figure 3. Changes over time of SARS-CoV-2 spike-specific CD4^+^ T-cells.**

(a) For spike-specific CD8^+^ T-cells, memory cells were gated based on the expression of 4-1BB and CD69. (b) Representative plots of granzyme A (GZMA), granzyme B (GZMB), and perforin expression in CD69^+^4-1BB^+^CD8^+^ memory T-cells. Expression of each cytotoxic molecule in CD8^+^ naïve T-cells was used for gating control.

**Supplementary Figure 4. Functional characteristics of SARS-CoV-2 spike-specific CD8^+^ T-cells.**

(a, b) Frequencies of spike-specific CD69^+^4-1BB^+^CD8^+^ T-cell subpopulations expressing a different combination of GZMA, GZMB, and perforin in healthy donors (black), CNI group (red), and CNI+other drug(s) group (blue) at pre- (a) and post-third boost (b). The response patterns are color-coded by the number of cytotoxic molecules in spike-specific CD69^+^4-1BB^+^CD8^+^ T cells (0: yellow, 1: green, 2: blue, 3: red). All experiments were performed once.

**Supplementary Figure 5. CD4^+^ T-cell responses against SARS-CoV-2 variants of concern induced by mRNA vaccine related to Figure 5.**

(a, c) Comparison of spike-specific CD154^+^CD4^+^ T-cell frequency against Wuhan-1, BA.5, BQ.1.1, and XBB spike peptides in CD4 total memory T-cells at (a) pre-and (c) post-third boost obtained from healthy donors and LTRs (HDs: black, CNI: red, CNI+other drug(s): blue). (b, d) Fold-change of spike-specific CD154^+^CD4^+^ T-cell frequency against variants of concern at (b) pre- and (d) post-third boost in each group relative to Wuhan-1. The minus symbol denotes increased resistance. Shades of blue represent an increase in fold change, with darker shades indicating a larger positive fold change. Conversely, shades of red denote a decrease, with darker shades signifying a larger negative fold change. *P*-values were calculated using the Wilcoxon matched-pairs signed rank test. (e, f) Comparison of spike-specific Th2 CD4^+^ T-cell frequency against Wuhan-1, BA.5, BQ.1.1, and XBB spike peptides in CD4^+^ total memory T-cells at (e) pre- and (f) post-third boost obtained from healthy donors and LTRs (HDs: black, CNI: red, CNI+other drug(s): blue). (g, h) Fold-change of spike-specific Th2 CD4^+^ T-cell frequency against variants of concern at (g) pre- and (h) post-third boost in each group relative to Wuhan-1. The minus symbol denotes increased resistance. Shades of blue represent an increase in fold change, with darker shades indicating a larger positive fold change. Conversely, shades of red denote a decrease, with darker shades signifying a larger negative fold change. *P*-values (two-sided) were calculated using the Wilcoxon matched-pairs signed rank test. Sample size, pre-3rd boost: 43 vs 21 vs 29, post-3rd boost: 43 vs 20 vs 29. All experiments were performed once.

**Supplementary Figure 6. Multivariate analysis of spike-specific CD8 T-cells in liver transplant recipients.**

(a) Multivariable logistic regression model (OR and 95% CI) for factors of the non-responder group (no increase in spike-specific CD8^+^ T-cells with the third boost) and responder group (increase in spike-specific CD8^+^ T-cells with the third boost).

**Supplementary Figure 7. Dose of steroids affects antibody responses but not CD4 and CD8 T-cell responses.**

(a) Correlation between dose of steroids and anti-RBD antibody endpoint titers (left panel), frequencies of CD154^+^CD4 T cells (middle panel), and frequencies of CD69^+^4-1BB^+^CD8 T cells (right panel) (n = 13). *P*-values (two-sided) were calculated using the Spearman’s rank test.
