# Supplementary material for: Humoral and cellular immune responses to COVID-19 mRNA vaccines in immunosuppressed liver transplant recipients
Source: Commun Med (Lond). 2024 Feb 26;4:30. doi: 10.1038/s43856-024-00448-4 (PMC10897323; doi:10.1038/s43856-024-00448-4)
Supplement: Supplementary file 3 — Description of Additional Supplementary Files [file 43856_2024_448_MOESM3_ESM.pdf]

Description of Additional Supplementary Files

File name: Supplementary Data 1
Description: Source Data.
