# Supplementary material for: Humoral and cellular immune responses to COVID-19 mRNA vaccines in immunosuppressed liver transplant recipients
Source: Commun Med (Lond). 2024 Feb 26;4:30. doi: 10.1038/s43856-024-00448-4 (PMC10897323; doi:10.1038/s43856-024-00448-4)
Supplement: Supplementary file 5 — Reporting Summary [file 43856_2024_448_MOESM5_ESM.pdf]

Corresponding author(s): Dr. Takuya Yamamoto

Last updated by author(s): 2023-12-02

Reporting Summary

Nature Portfolio wishes to improve the reproducibility of the work that we publish. This form provides structure for consistency and transparency in reporting. For further information on Nature Portfolio policies, see our Editorial Policies and the Editorial Policy Checklist.

Please do not complete any field with "not applicable" or n/a. Refer to the help text for what text to use if an item is not relevant to your study.
For final submission: please carefully check your responses for accuracy; you will not be able to make changes later.

Statistics

For all statistical analyses, confirm that the following items are present in the figure legend, table legend, main text, or Methods section.

n/a | Confirmed

- The exact sample size (*n*) for each experimental group/condition, given as a discrete number and unit of measurement
- A statement on whether measurements were taken from distinct samples or whether the same sample was measured repeatedly
- The statistical test(s) used AND whether they are one- or two-sided
  Only common tests should be described solely by name; describe more complex techniques in the Methods section.
- A description of all covariates tested
- A description of any assumptions or corrections, such as tests of normality and adjustment for multiple comparisons
- A full description of the statistical parameters including central tendency (e.g. means) or other basic estimates (e.g. regression coefficient) AND variation (e.g. standard deviation) or associated estimates of uncertainty (e.g. confidence intervals)
- For null hypothesis testing, the test statistic (e.g. *F*, *t*, *r*) with confidence intervals, effect sizes, degrees of freedom and *P* value noted
  Give P values as exact values whenever suitable.
- For Bayesian analysis, information on the choice of priors and Markov chain Monte Carlo settings
- For hierarchical and complex designs, identification of the appropriate level for tests and full reporting of outcomes
- Estimates of effect sizes (e.g. Cohen's *d*, Pearson's *r*), indicating how they were calculated

Our web collection on statistics for biologists contains articles on many of the points above.

Software and code

Policy information about availability of computer code

Data collection: ELISA using Epoch 2 Microplate Spectrophotometer (Agilent, Santa Clara, CA, USA). Luciferase assay for neutralizing activity using EnSpire

Data analysis: FlowJo Software version 10.8.1. (BD, Ashland, OR) was used for analyzing flow cytometry data. Statistical analyses were performed with

For manuscripts utilizing custom algorithms or software that are central to the research but not yet described in published literature, software must be made available to editors and reviewers. We strongly encourage code deposition in a community repository (e.g. GitHub). See the Nature Portfolio guidelines for submitting code & software for further information.

Data

Policy information about availability of data

All manuscripts must include a data availability statement. This statement should provide the following information, where applicable:
- Accession codes, unique identifiers, or web links for publicly available datasets
- A description of any restrictions on data availability
- For clinical datasets or third party data, please ensure that the statement adheres to our policy

Datasets generated and/or analyzed during the current study are available in the paper or are appended as Supplementary Data 1. The data supporting the findings of this study are available from the corresponding author upon request.

Human research participants

Policy information about studies involving human research participants and Sex and Gender in Research.

Reporting on sex and gender	26 females and 28 males in liver transplant recipient group.
Population characteristics	Donor characteristics are reported in Table 1.
Recruitment	A total of 98 individuals (44 healthy donors and 54 LTRs) were recruited from Osaka University, Japan.
Ethics oversight	The study protocol and procedures were reviewed and approved by the Institutional Ethics Committees of the National

Note that full information on the approval of the study protocol must also be provided in the manuscript.

Field-specific reporting

Please select the one below that is the best fit for your research. If you are not sure, read the appropriate sections before making your selection.

(x) Life sciences ( ) Behavioural & social sciences ( ) Ecological, evolutionary & environmental sciences

Life sciences study design

All studies must disclose on these points even when the disclosure is negative.

Sample size	A total of 98 individuals (44 healthy donors and 54 LTRs) were recruited from Osaka University, Japan.
Data exclusions	All samples with available data were included in the analyses.
Replication	All assays were performed by single acquisition for each condition.
Randomization	Randomization was not relevant to this study as this was an observational study.
Blinding	Blinding was not relevant to this study because of the observational design.

Behavioural & social sciences study design

All studies must disclose on these points even when the disclosure is negative.

Study description	
Research sample	
Sampling strategy	
Data collection	
Timing	
Data exclusions	
Non-participation	
Randomization	

Ecological, evolutionary & environmental sciences study design

All studies must disclose on these points even when the disclosure is negative.

Study description	
Research sample	
Sampling strategy	
Data collection	
Timing and spatial scale	
Data exclusions	
Reproducibility	
Randomization	

Blinding

Did the study involve field work? ○ Yes ○ No

Field work, collection and transport

Field conditions

Location

Access & import/export

Disturbance

Reporting for specific materials, systems and methods

We require information from authors about some types of materials, experimental systems and methods used in many studies. Here, indicate whether each material, system or method listed is relevant to your study. If you are not sure if a list item applies to your research, read the appropriate section before selecting a response.

Materials & experimental systems

n/a	Involved in the study
	Antibodies
	Eukaryotic cell lines
●	Palaeontology and archaeology
●	Animals and other organisms
●	Clinical data
●	Dual use research of concern

Methods

n/a	Involved in the study
●	ChIP-seq
	Flow cytometry
●	MRI-based neuroimaging

Antibodies

Antibodies used: For ELISA assay, biotin mouse anti-human IgG (BD Biosciences, cat. 555785) was used.

Validation: All antibodies were commercially available.

Eukaryotic cell lines

Policy information about cell lines and Sex and Gender in Research

Cell line source(s): HEK-293A cells expressing ACE2 and TMPRSS2 were obtained as follow. HEK293A cells (American Type Culture Collection

Authentication: not authenticated

Mycoplasma contamination: not tested

Commonly misidentified lines (See ICLAC register): No commonly misidentified cell lines used.

Palaeontology and Archaeology

Specimen provenance

Specimen deposition

Dating methods

☐ Tick this box to confirm that the raw and calibrated dates are available in the paper or in Supplementary Information.

Ethics oversight

Note that full information on the approval of the study protocol must also be provided in the manuscript.

Animals and other research organisms

Policy information about studies involving animals; ARRIVE guidelines recommended for reporting animal research, and Sex and Gender in Research

Laboratory animals

Wild animals

Reporting on sex

Field-collected samples

Ethics oversight

Note that full information on the approval of the study protocol must also be provided in the manuscript.

Clinical data

Policy information about clinical studies

All manuscripts should comply with the ICMJE guidelines for publication of clinical research and a completed CONSORT checklist must be included with all submissions.

Clinical trial registration

Study protocol

Data collection

Outcomes

Dual use research of concern

Policy information about dual use research of concern

Hazards

Could the accidental, deliberate or reckless misuse of agents or technologies generated in the work, or the application of information presented in the manuscript, pose a threat to:

No	Yes	
○	○	Public health
○	○	National security
○	○	Crops and/or livestock
○	○	Ecosystems
○	○	Any other significant area

Experiments of concern

Does the work involve any of these experiments of concern:

No	Yes	
○	○	Demonstrate how to render a vaccine ineffective
○	○	Confer resistance to therapeutically useful antibiotics or antiviral agents
○	○	Enhance the virulence of a pathogen or render a nonpathogen virulent
○	○	Increase transmissibility of a pathogen
○	○	Alter the host range of a pathogen
○	○	Enable evasion of diagnostic/detection modalities
○	○	Enable the weaponization of a biological agent or toxin
○	○	Any other potentially harmful combination of experiments and agents

ChIP-seq

Data deposition

- [ ] Confirm that both raw and final processed data have been deposited in a public database such as GEO.
- [ ] Confirm that you have deposited or provided access to graph files (e.g. BED files) for the called peaks.

Data access links
May remain private before publication.

Files in database submission

Genome browser session
(e.g. UCSC)

Methodology

Replicates

Sequencing depth

Antibodies

Peak calling parameters

Data quality

Software

Flow Cytometry

Plots

Confirm that:

- [x] The axis labels state the marker and fluorochrome used (e.g. CD4-FITC).
- [x] The axis scales are clearly visible. Include numbers along axes only for bottom left plot of group (a 'group' is an analysis of identical markers).
- [x] All plots are contour plots with outliers or pseudocolor plots.
- [x] A numerical value for number of cells or percentage (with statistics) is provided.

Methodology

Sample preparation	Blood samples were collected, and peripheral blood mononuclear cells (PBMCs) were isolated via density gradient
Instrument	BD FACSymphony A5 flow cytometer (BD Biosciences, San Jose, CA)
Software	FlowJo Software version 10.8.1.
Cell population abundance	Cells were not enriched prior to in vitro stimulation.
Gating strategy	The gating strategy is included as Supplementary Figures. After gating live single T-cells based on the forward scatter area and

- [x] Tick this box to confirm that a figure exemplifying the gating strategy is provided in the Supplementary Information.

Magnetic resonance imaging

Experimental design

Design type

Design specifications

Behavioral performance measures

Acquisition

Imaging type(s)

Field strength

Sequence & imaging parameters

Area of acquisition

Diffusion MRI ○ Used ○ Not used

Preprocessing

Preprocessing software

Normalization

Normalization template

Noise and artifact removal

Volume censoring

Statistical modeling & inference

Model type and settings

Effect(s) tested

Specify type of analysis: ○ Whole brain ○ ROI-based ○ Both

Statistic type for inference
(See Eklund et al. 2016)

Correction

Models & analysis

n/a	Involved in the study
☐	☐ Functional and/or effective connectivity
☐	☐ Graph analysis
☐	☐ Multivariate modeling or predictive analysis

Functional and/or effective connectivity

Graph analysis

Multivariate modeling and predictive analysis

This checklist template is licensed under a Creative Commons Attribution 4.0 International License, which permits use, sharing, adaptation, distribution and reproduction in any medium or format, as long as you give appropriate credit to the original author(s) and the source, provide a link to the Creative Commons license, and indicate if changes were made. The images or other third party material in this article are included in the article's Creative Commons license, unless indicated otherwise in a credit line to the material. If material is not included in the article's Creative Commons license and your intended use is not permitted by statutory regulation or exceeds the permitted use, you will need to obtain permission directly from the copyright holder. To view a copy of this license, visit http://creativecommons.org/licenses/by/4.0/
